# PPOS Protocol Effectively Improves the IVF Outcome Without Increasing the Recurrence Rate in Early Endometrioid Endometrial Cancer and Atypical Endometrial Hyperplasia Patients After Fertility Preserving Treatment

**DOI:** 10.3389/fmed.2021.581927

**Published:** 2021-07-27

**Authors:** Jiazhou Chen, Yali Cheng, Wei Fu, Xiandong Peng, Xiaoxi Sun, Hua Chen, Xiaojun Chen, Min Yu

**Affiliations:** ^1^Shanghai JIAI Genetics and IVF Institute, Obstetrics and Gynecology Hospital of Fudan University, Shanghai, China; ^2^Shanghai Key Laboratory of Female Reproductive and Endocrine-Related Diseases, Shanghai, China; ^3^Department of Gynecologic Oncology, Obstetrics and Gynecology Hospital of Fudan University, Shanghai, China

**Keywords:** endometrial diseases, ovulation Induction, frozen embryo transfer, recurrence, progestin primed ovarian stimulation protocol, *in vitro* fertilization, atypical hyperplasia

## Abstract

**Objective:** To investigate the effectiveness and recurrence risk of different ovulation stimulation protocols in early-stage endometrioid endometrial cancer (EEC) and atypical endometrial hyperplasia (AEH) patients after successful fertility preserving treatment.

**Design:** A retrospective review of clinical files between June 2012 and July 2018.

**Setting:** University hospital.

**Patients:** Ninety seven women (74 AEH and 23 early-stage EEC patients) underwent *in vitro* fertilization (IVF) and frozen-thawed embryo transfer (FET) after successful fertility preserving treatment. All patients received megestrol acetate which was initiated immediately after AEH or EEC diagnosis by hysteroscopy. Fertility treatment was initiated after confirmation of complete response by two consecutive hysteroscopic evaluations and endometrium biopsy in a 3-month interval. Women with tubal factors underwent IVF treatment directly. Women who failed to conceive spontaneously within 12 months or after other infertility treatments like ovulation induction for 6 consecutive months or 2 consecutive artificial insemination failures were also offered IVF treatment.

**Main Outcome Measure (s):** The clinical and laboratory embryo data, clinical pregnancy outcomes and endometrial disease recurrence rates.

**Results:** Compared with the standard regimen group, the good-quality embryo rate was higher in progestin primed ovarian stimulation (PPOS) regimen group (*P* = 0.034). Univariate analysis showed significant differences in age (*P* = 0.033), treatment time of endometrial lesions (*P* < 0.001), and duration of Gn treatment (*P* = 0.018) between the recurrent and non-recurrent groups. In the adjusted model of multivariate logistic regression analysis, the age (*P* = 0.014) at ovulation induction and treatment time of endometrial lesions (*P* < 0.001) were significantly correlated with the recurrence of endometrial disease.

**Conclusions:** The PPOS protocol is a feasible and safe strategy to stimulate ovulation during IVF after fertility preservation therapy, and the age at ovulation induction and treatment time of endometrial lesions are two stable predictors of recurrence in endometrial diseases.

## Introduction

Endometrioid endometrial cancer (EEC) is mostly associated with long-lasting estrogen exposure without progesterone protection due to ovulation disorders. Atypical endometrial hyperplasia (AEH) is a premalignant condition which can develop into EEC in a couple of years if it is not treated properly (1). The primary therapeutic option for early-stage EEC or AEH is hysterectomy. Nevertheless, fertility preserving treatment is an optional choice for certain young patients with early-stage EEC or AEH who strongly desire to preserve their fertility.

Although the response rate of fertility preserving treatment of EEC and AEH patients is relatively satisfactory (80–90%), the pregnancy rate and live birth rate of these patients are still unsatisfactory (2, 3). Several factors including disease recurrence (4), damage of normal endometrium (3), and increased body mass (5) are responsible for the low pregnancy and birth rates observed in these patients. The combination of *in vitro* fertilization (IVF) and frozen-thawed embryo transfer (FET) is among the main methods for improving the pregnancy rate of EEC and AEH patients (6). Ovarian induction is an essential part of IVF procedure that allows the screening of high-quality embryos for transfer. Ovulation stimulation protocols used in IVF after successful fertility preserving treatment include conventional short agonist regimen and antagonist regimen (standard regimen), mild stimulation protocol [usually clomiphene citrate (CC) or letrozol (LE) combined with gonadotropins (Gn)] and progestin primed ovarian stimulation (PPOS) protocol (7). However, there are two major concerns regarding ovarian induction in EEC and AEH patients. First, because most EEC and AEH patients suffer from ovarian dysfunction, it is not clear which kind of ovarian stimulation protocol is the most effective for these patients. Secondly, and most importantly, ovulation induction during infertility treatment may stimulate the ovarian production of 17β-estradiol (E_2_), which may promote the progression of EEC and AEH or induce their recurrence (8, 9). Due to the relatively limited number of EEC and AEH patients receiving ovarian stimulation, there is no report on the effectiveness and recurrence risk of different ovarian stimulation protocols in EEC and AEH patients.

Herein, a retrospective study based on a relatively large number of patients (including 74 early-stage EEC and 23 AEH patients) who underwent fertility-sparing treatment was performed to explore the feasibility and safety of different ovulation stimulation protocols. We compared the stimulating characteristics and outcomes of each ovulation induction regimen, and focused on the assessment of prognostic factors for the recurrence risk of endometrial disease.

## Materials and Methods

### Patients

**Figure 1:**
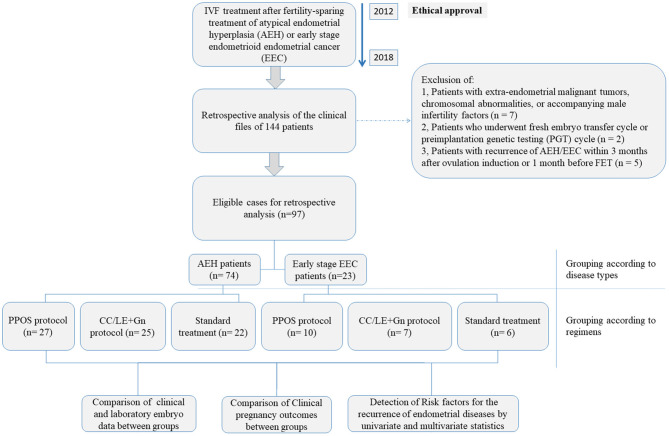
Flowchart describing the design of the current retrospective study.

This retrospective study was carried out based on the clinical data of 144 patients who were reffered to the Shanghai JiAi Genetics & IVF Institute affiliated to Obstetrics and Gynecology Hospital of Fudan University between June 2012 and July 2018 for IVF treatment after fertility-sparing treatment of atypical endometrial hyperplasia (AEH) or early stage endometrioid endometrial cancer (EEC). After exclusion of: (1) patients with extra-endometrial malignant tumors, chromosomal abnormalities, or accompanying male infertility factors (*n* = 7), (2) patients who underwent fresh embryo transfer cycle or preimplantation genetic testing (PGT) cycle (*n* = 2) and (3) patients with recurrence of AEH/EEC within 3 months after ovulation induction or 1 month before FET (*n* = 5), 97 patients who underwent IVF treatment after fertility-sparing treatment of AEH (*n* = 74) or well-differentiated early-stage EEC (*n* = 23, International Federation of Gynecology and Obstetrics (FIGO) stage IA, without myometrial invasion and extrauterine metastases) were finally included in this study and their clinical and laboratory data were retrospectively analyzed. The overall design of the current study was shown in Figure 1. Pathological diagnosis of endometrial biopsy under hysteroscopy was confirmed in all patients in Obstetrics and Gynecology Hospital of Fudan University. According to the conventional guidelines in China, patients diagnosed with early-stage endometrioid endometrial cancer (EEC) or atypical endometrial hyperplasia (AEH) should undergo total hysterectomy under informed consent agreement. Therefore, before the start of IVF treatment, ethical approval was obtained from the Ethics Committees of Obstetrics and Gynecology Hospital of Fudan University (number JIAI E 2012-02). All patients were fully aware of the risks of conservative treatment and IVF treatment for AEH and EEC. All patients signed an informed consent to use their clinical and pathological data for research purpose. Details on each treatment approach and different protocols were as described below.

### Conservative Treatment

Conservative treatment and evaluation of therapeutic effects were described in a previous study (10). Briefly, megestrol acetate treatment (160 mg po qd) was initiated immediately after AEH or EEC diagnosis by hysteroscopy. Comprehensive evaluation was performed under hysteroscopy every 3 months during treatment till complete response.

### Fertility Treatment

Fertility treatment was initiated after confirmation of complete response by two consecutive hysteroscopic evaluations and endometrium biopsies in a 3-month interval. Women with tubal diseases underwent IVF treatment directly. Women who failed to conceive spontaneously within 12 months or after other infertility treatments like ovulation induction for 6 consecutive months or 2 consecutive artificial insemination failures were also offered IVF treatment. Women with extra-endometrial malignant tumors, chromosomal abnormalities, or accompanying male infertility factors were excluded. Fresh embryo transfer cycle and preimplantation genetic testing (PGT) cycle were also excluded. All patients underwent hysteroscopic endometrial biopsy within 3 months after ovulation induction and 1 month before FET to exclude the recurrence of AEH and EEC.

### Ovulation Stimulation Regimens

For all patients, endometrial hysteroscopic biopsy was taken prior to FET. Since we planned to follow up the recurrence rate of endometrial lesions after ovulation induction, the patients were subjected to the ovulation induction regimens with shorter treatment period, which included: PPOS, mild stimulation protocols, standard regimen and short agonist regimen. The appropriate regimen for ovarian stimulation was chosen by experienced doctors based on comprehensive consideration of each patient's basic hormone levels, BMI (body mass index), AFC (antral follicle count), drug compliance and financial affordability. The patients in PPOS protocol were administered 20 mg/d oral dydrogesterone (DYG) (Duphaston; Abbott Biologicals B.V., Netherlands) and human menopausal gonadotropin (HMG) (Lizhu Pharmaceutical Trading Co., Zhuhai, China) injection at a dose of 150–300 international unit (IU) daily from day 2/3 of the menstrual cycle to the day of trigger. Compared to medroxyprogesterone acetate (MPA, another progesterone in PPOS regimen), dydrogesterone showed a similar profile to natural progesterone (11); in light of this, dydrogesterone was used for patient treatment. The mild stimulation protocol was performed as follows: starting from day 3 of the menstrual cycle, patients received oral CC (Codal Synto Ltd., Cyprus) at 100 mg/d or LE (FuRui, Hengrui Ltd, China) at 10 mg/d and HMG/r-FSH (Gonal-f) (Merck Serono SA Aubonne Branch) injection at 150–300 IU daily. Standard protocol included short agonist regimen and antagonist regimen. Short agonist regimen was performed from day 2/3 of the menstrual cycle and consisted of an injection of 0.1 mg/day of GnRH-a (Decapeptyl, Ferring, Switzerland) which was followed by the injection of 150–300 IU/day of Gn from the next day. For the antagonistic regimen, the fixed scheme was adopted, and the antagonists-Cetrotide (Merck Serono SA Aubonne Branch) was administered on the 6th day of Gn use. In all protocols, the dosage of Gn was adjusted according to the level of estrogen and follicular growth.

### Oocyte Retrieval, Embryo Culture and FET

Ovulation was induced with 5,000 to 10,000 IU of human chorionic gonadotropin (hCG) (Livzon/China) according to the patient's body weight and peak E_2_ level when the diameter of at least one follicle was >18 mm (12). Oocyte retrieval was carried out 35 h after hCG trigger and conventional IVF was performed for most patients, and intracytoplasmic sperm injection (ICSI) was performed. Following the criteria judged by Cummins et al. (13), the symmetry or regularity of the blastomere, the cytoplasmic quality, and the fragmentary degree of embryo were checked on day 3 after oocyte retrieval, and all good-quality embryos (grade I and II 8-cell embryos) were cryopreserved by vitrification whereas the embryos in grade III and IV needed to be expanded by culture until they enter the blastocyst stage. Then, Gardner scoring was used to select eligible blastocysts (not <3BC grade) for vitrification on day 5/6 (14). Thawing was carried out on the day of the blastocyst transfer. The cryoloop ring was removed from the liquid nitrogen and placed in the air for 5 s before being immersed in recovery solutions. Next, the laser-assisted incubation of fully recovered blastocysts was performed and then the blastocysts were transferred to the blastocyst culture medium (Quinn's, SAGE, USA). A detailed description of FET procedure was described in our previous publications (15). In brief, the FET process was executed in a natural or induced menstrual cycle. The patient underwent routine ultrasound examinations and oral estradiol valerate (Sigma, St. Louis, Missouri, USA) on day 2 to day 4 of menstruation. The endometrium was monitored by ultrasound, and when its thickness reached 7 mm, 40 mg qd progesterone was injected intramuscularly. Embryo transfer was conducted under ultrasonic guidance, with only one or two embryos implanted per patient at a time. After transplantation, progesterone vaginal gel (Crinone, Merck Serono, Germany) was given to support the luteal phase.

### Pregnancy Test

Serum β-hCG test was performed on the 14th day after FET using the total β-hCG kit (Beckman Coulter/USA) to confirm the occurrence of pregnancy. Serum β-hCG higher than 100 mIU/mL indicated biochemical pregnancy and ultrasound was used to detect yolk sac 4 weeks after FET was defined as clinical pregnancy (16).

### Follow-Up and Outcomes Assessment

Since most of the pregnancies and recurrences occur in the first 2 years (3), our follow-up time was 24 months, which was initiated after the ovarian stimulation. None of the patients were lost during the 2-year follow-up, therefore; all 97 patients were included in the univariate or multivariate analysis of risk factors for the recurrence of endometrial disease. We retrospectively evaluated the basic information of each patient, clinical and laboratory embryo data, clinical pregnancy outcomes, and pathological report of hysteroscopy biopsy after treatment. The baseline characteristics of patient included age, anti-mullerian hormone (AMH), body mass index (BMI), homeostasis model assessment insulin resistance (HOMA-IR) index (calculated by fasting blood glucose (FBG; mmol/L) × fasting insulin (FINS; μU/mL)/22.5), lesion types, treatment time of endometrial lesions, and IVF interval time. The effectiveness of ovarian stimulation regimens was evaluated by the E_2_ level on hCG trigger day, Gn dosage, duration of Gn treatment, the number of oocytes obtained, MII oocytes rate, fertility rate, good-quality embryo rate, FET cycle cancellation rate, thickness of endometrium, and pregnancy rate. The safety of ovarian stimulation regimens was assessed by comparing the recurrence rate of endometrial lesions.

### Statistical Analysis

Statistical analysis was performed using SPSS 25.0 software (IBM Corp., Armonk, NY, USA). Quantitative variables were expressed as means ± standard deviation and categorical variables were described using frequencies and proportions. For quantitative variables, the Shapiro-Wilk test and Levene test were used to evaluate their normal distribution and variance homogeneity, respectively. The Chi-squared test was used to analyze categorical variables. For quantitative variables, the Kruskal-Wallis test was used to analyze the differences among three or more groups, and comparison between two groups was executed by the Mann-Whitney-Wilcoxon test or unpaired *t*-test. Binary logistic regression was applied to explore the risk factors on the recurrence of endometrial diseases, and receiver operating characteristic (ROC) curve was used to evaluate the diagnostic accuracy. *P* < 0.05 was considered to be statistically significant. Before the retrospective analysis, sample size was calculated using Raosoft sample size calculator (http://www.raosoft.com/samplesize.html) to detect the minimum number of patients to be included. Assuming a confidence level of 90%, a margin of error of 5% and a response distribution of 50%, the minimum sample size was determined to be 89 eligible patients.

## Results

### Baseline Characteristics of Patient in Different Ovulation Stimulation Regimens

A total of 97 patients (23 cases diagnosed with early-stage EEC and 74 with AEH) who had undergone different ovulation stimulation regimens were included in this study. Only the first oocyte retrieval cycle of each patient was included in the retrospective analysis; we collected 37 cycles in PPOS protocol, 32 cycles in mild stimulation protocol (CC/LE+Gn), and 28 cycles in standard protocol. The basic information of patient characteristics in different ovulation stimulation regimens was shown in Table 1. There was no significant difference in age, HOMA-IR, lesion types, treatment time of endometrial lesions, and IVF interval time among the three different ovulation stimulation regimens (all *P* > 0.05, Table 1). The three groups significantly differed in AMH (*P* = 0.004) and BMI (*P* = 0.002). Specifically, the AMH of PPOS (*P* = 0.027) and standard regimen (*P* = 0.009) groups was higher than that of CC/LE+Gn regimen group, and the BMI decreased significantly in CC/LE+Gn regimen group when compared with the standard regimen groups (*P* = 0.018). However, no significant difference in baseline characteristics of patient was observed between the PPOS and standard regimen groups (Table 1).

**Table 1:** Baseline characteristics of patient in different ovulation stimulation regimens.

**Characteristic**	**PPOS (*n* = 37)**	**CC/LE+Gn (*n* = 32)**	**Standard regimen (*n* = 28)**	***P*-value**
Age (years)	31.43 ± 4.01	32.75 ± 3.84	31.75 ± 3.91	0.354^#^
AMH (ng/mL)	3.67 ± 2.83^a^	2.60 ± 3.98^b^	4.54 ± 4.10	0.004^**#**^
BMI (kg/m^2^)	23.91 ± 3.69	22.10 ± 3.44^b^	25.30 ± 3.23	0.002^#^
HOMA-IR	3.17 ± 2.42	2.89 ± 2.41	3.00 ± 1.77	0.630^#^
Number of lesion types				0.833^$^
Atypical	27	25	22	
Cancer	10	7	6	
Treatment time of endometrial lesions (months)	7.35 ± 5.34	7.68 ± 5.81	7.71 ± 6.20	0.995^#^
IVF interval time (months)	5.89 ± 3.42	7.06 ± 4.65	5.75 ± 3.69	0.456^#^

*PPOS, progestin primed ovarian stimulation; CC, clomiphene citrate; LE, letrozol; Gn, gonadotropin; AMH, anti-mullerian hormone; BMI, body mass index; HOMA-IR, Homeostasis model assessment of insulin resistance; IVF, in vitro fertilization*.

# *Kruskal-Wallis test*.

$ *Chi-squared test*.

a *P < 0.05, PPOS vs. CC/LE+Gn*.

b *P < 0.05, CC/LE+Gn vs. Standard regimen*.

### Stimulation Characteristics and Outcomes for Each Ovulation Induction Treatment Group

As shown in Table 2, compared to the standard regimen group, the content of E_2_ level on hCG trigger day (*P* = 0.003) and the number of oocytes retrieved (*P* < 0.001) were significantly lower in the CC/LE+Gn regimen group. Moreover, the results showed that compared to the standard regimen group (0.72 ± 0.31%), the good-quality embryo rate was significantly increased in PPOS regimen group (0.89 ± 0.26%) (*P* = 0.034, Table 2). In addition, the pregnancy rate (40.54%) of PPOS regimen group was significantly higher than that of the CC/LE+Gn regimen group (9.38%; *P* = 0.023), but the difference between PPOS and the standard regimen groups (21.43%) was not statistically significant (*P* = 0.259, Table 2). However, there was no significant difference in Gn dosage, duration of Gn treatment, MII oocytes rate, fertility rate, FET cycle cancellation rate, and recurrence rate among the different ovulation stimulation regimens (all *P* > 0.05, Table 2). These results indicated that patients in PPOS protocol had a better response to ovulation induction without increasing the recurrence rate.

**Table 2:** Stimulation characteristics and outcomes for each ovulation induction treatment group.

**Characteristic**	**PPOS (*n* = 37)**	**CC/LE+Gn (*n* = 32)**	**Standard regimen (*n* = 28)**	***P*-value**
E2 level on hCG trigger day (pg/mL)	3163.78 ± 1821.38	2287.66 ± 2184.09^c^	3866.75 ± 2188.61	0.003^#^
Gn dosage (vials^Ψ^)	33.72 ± 15.84	31.06 ± 15.61	29.07 ± 15.66	0.298^#^
Duration of Gn treatment (days)	9.62 ± 2.41	9.19 ± 2.66	9.46 ± 3.01	0.906^#^
The number of oocytes retrieved	8.49 ± 4.36	6.38 ± 6.75^c^	13.46 ± 8.13	< 0.001^#^
MII oocytes rate	0.89 ± 0.13	0.93 ± 0.11	0.90 ± 0.10	0.704^#^
Fertility rate	0.88 ± 0.16	0.91 ± 0.15	0.87 ± 0.13	0.527^#^
Good-quality embryo rate	0.89 ± 0.26^b^	0.75 ± 0.33	0.72 ± 0.31	0.024^#^
FET cycle cancellation rate	9 (24.32%)	16 (50.00%)	9 (32.14%)	0.077^$^
Thickness of endometrium (mm)	9.15 ± 2.01	8.75 ± 1.34	8.68 ± 1.38	0.866^#^
Pregnancy rate	15 (40.54%)^a^	3 (9.38%)	6 (21.43%)	0.010^$^
Recurrence rate	6 (16.22%)	7 (21.88%)	5 (17.86%)	0.828^$^

*PPOS, progestin primed ovarian stimulation; CC, clomiphene citrate; LE, letrozol; Gn, gonadotropin; E2, 17β-estradiol; hCG, human chorionic gonadotropin; FET, frozen-thawed embryo transfer*.

# *Kruskal-Wallis test*.

$ *Chi-squared test*.

a *P < 0.05, PPOS vs. CC/LE+Gn*.

b *P < 0.05, PPOS vs. Standard regimen*.

c *P < 0.05, CC/LE+Gn vs. Standard regimen*.

Ψ *Eachvial contains 75 IU (international unit) of gonadotropin*.

### Univariate Analysis of Risk Factors With the Recurrence of Endometrial Diseases

As shown in Table 3, 18 cases were diagnosed with the recurrence of endometrial disease, and 79 cases were diagnosed with no recurrence of endometrial disease. Univariate analysis showed significant differences in age, treatment time of endometrial lesions, and duration of Gn treatment between the recurrence and non-recurrence groups (*P* < 0.05, Table 3). The age (33.94 ± 4.45 years) and treatment time of endometrial lesions (12.94 ± 6.93 months) in the recurrence group were significantly higher than those in the non-recurrence group (31.51 ± 3.68 years and 6.34 ± 4.61 months, respectively), while the duration of Gn treatment (7.94 ± 2.35 days) was significantly lower than that of the non-recurrence group (9.77 ± 2.58 days). No significant difference in other indicators such as AMH, BMI, HOMA-IR, ovulation induction regimens, lesion types, IVF interval time, E_2_ level on hCG trigger day and Gn dosage was observed between the recurrence and non-recurrence groups (*P* > 0.05, Table 3).

**Table 3:** Univariate analysis of risk factors with the recurrence of endometrial diseases.

**Variables**	**Recurrence**	**Non-recurrence**	***P*-value**
	**(*n* = 18)**	**(*n* = 79)**	
Age (years)	33.94 ± 4.45	31.51 ± 3.68	0.033^&^
AMH (ng/mL)	3.65 ± 3.78	3.55 ± 3.67	0.900^&^
BMI (kg/m^2^)	24.48 ± 4.12	23.54 ± 3.57	0.378^@^
HOMA-IR	3.31 ± 2.11	2.97 ± 2.26	0.303^&^
Number of patients for ovulation induction regimens	0.828^$^		
PPOS	6	31	
CC/LE+Gn	7	25	
Standard regimen	5	23	
Number of lesion types		0.093^$^	
Atypical	11	63	
Cancer	7	16	
Treatment time of endometrial lesions (months)	12.94 ± 6.93	6.34 ± 4.61	<0.001^&^
IVF interval time (months)	7.00 ± 4.00	6.06 ± 3.93	0.261^&^
E2 level on hCG trigger day (pg/mL)	3510.78 ± 1865.51	2978.99 ± 2180.94	0.165^&^
Gn dosage (vials^Ψ^)	28.08 ± 14.60	32.28 ± 15.88	0.385^&^
Duration of Gn treatment (days)	7.94 ± 2.53	9.77 ± 2.58	0.018^&^

*AMH, anti-mullerian hormone; BMI, body mass index; HOMA-IR, Homeostasis model assessment of insulin resistance; PPOS, progestin primed ovarian stimulation; CC, clomiphene citrate; LE, letrozol; IVF, in vitro fertilization; hCG, human chorionic gonadotropin; E_2_, 17β-estradiol; Gn, gonadotropin*.

& *Mann-Whitney-Wilcoxon test*.

@ *Unpaired t-test*.

$ *Chi-squared test*.

Ψ *Each vial contains 75 IU (international unit) of gonadotropin*.

### Multivariate Logistic Regression Analysis of Risk Factors Associated With the Recurrence of Endometrial Diseases

We further performed multivariate logistic regression analysis to investigate the risk factors for the recurrence of endometrial disease. The results of unadjusted model showed that the age (aOR: 1.316, 95% CI: 1.06–1.633, and *P* = 0.013), treatment time of endometrial lesions (aOR: 1.282, 95% CI: 1.09–1.509, and *P* = 0.003), E_2_ level on hCG trigger day (aOR: 1.001, 95% CI: 1–1.001, and *P* = 0.015) and duration of Gn treatment (aOR: 0.547, 95% CI: 0.319–0.939, and *P* = 0.029) were significantly associated with the endometrial disease recurrence (*P* < 0.05, Table 4). Nevertheless, there were no significant correlations between disease recurrence and other indicators such as AMH, BMI, HOMA-IR, lesion types, IVF interval time, ovulation induction regimens and Gn dosage (*P* > 0.05, Table 4). Furthermore, each parameter was conditionally filtered to obtain an adjusted model, including age, BMI, treatment time of endometrial lesions, E_2_ level on hCG trigger day, and duration of Gn treatment. As shown in Table 4, in the adjusted model, the ovulation induction age (aOR: 1.228, 95% CI: 1.043–1.447, and *P* = 0.014) and treatment time of endometrial lesions (aOR: 1.236, 95% CI: 1.106–1.381, and *P* < 0.001) were significantly correlated with the recurrence of endometrial disease, and the established model had an area under ROC curve of 0.826 (*P* < 0.001).

**Table 4:** Multivariate logistic regression analysis of risk factors associated with the recurrence of endometrial diseases.

**Variables**	**Coeffcient (β)**	**Wals (χ^2^)**	**aOR (95%CI)**	***P*-value**
Unadjusted model				
Age	0.274	6.216	1.316 (1.06–1.633)	0.013
AMH	−0.101	0.524	0.904 (0.687–1.189)	0.469
BMI	0.278	2.410	1.321 (0.93–1.876)	0.121
HOMA-IR	0.072	0.098	1.075 (0.685–1.685)	0.754
Lesion types	0.039	0.002	1.04 (0.187–5.784)	0.965
Treatment time of endometrial lesions (months)	0.249	8.954	1.282 (1.09–1.509)	0.003
IVF interval time	0.132	1.519	1.141 (0.925–1.406)	0.218
Ovulation induction regimens				
Standard regimen		2.250		0.325
PPOS	1.359	1.390	3.891 (0.406–37.237)	0.238
CC/LE+Gn	1.811	2.233	6.119 (0.569–65.849)	0.135
E_2_ level on hCG trigger day	0.001	5.951	1.001 (1–1.001)	0.015
Gn dosage	0.015	0.106	1.015 (0.928–1.11)	0.745
Duration of Gn treatment (days)	−0.604	4.797	0.547 (0.319–0.939)	0.029
Adjusted model				
Age	0.206	6.069	1.228 (1.043–1.447)	0.014
Treatment time of endometrial lesions (months)	0.212	13.895	1.236 (1.106–1.381)	<0.001

*OR, odds ratio; CI, confidence interval; AMH, anti-mullerian hormone; BMI, body mass index; HOMA-IR, Homeostasis model assessment of insulin resistance; IVF, in vitro fertilization; PPOS, progestin primed ovarian stimulation; CC, clomiphene citrate; LE, letrozol; hCG, human chorionic gonadotropin; E_2_, 17β-estradiol; Gn, gonadotropin*.

*The variables in the adjusted model are adjusted for factors in the model using the forward conditional method. These confounding factors were excluded after adjustment. Age and treatment time of endometrial lesions are included in the logistic regression equation as: logit P = −10.109 + 0.206x_1_ + 0.212x_2_. The −2 log likelihood = 68.716; the Nagelkerke R^2^ = 0.360*.

## Discussion

In the present study, our results showed that the PPOS regimen is a feasible and safe strategy for stimulating ovulation during IVF after fertility preservation therapy. Compared with the standard regimen group, it promoted the good-quality embryo rate without increasing the recurrence rate in early-stage EEC and AEH patients. Moreover, it was found that the age of ovulation induction and treatment time of endometrial lesions were two stable prognostic factors for the recurrence of endometrial disease. To the best of our knowledge, this retrospective study on the IVF outcome of fertility preserving patients with EEC and AEH involved most patients so far.

The key concern of IVF treatment in EEC and AEH patients is the recurrence of endometrial disease. Every exposure to a high estrogen environment increases the risk of recurrence; thus, the ovulation induction efficiency is of great importance. Malmusi et al. (17) found that the pregnancy outcomes of the mild stimulation regimen were similar to the gonadotropin-releasing hormone (GnRH) agonist regimen. In addition, other previous studies obtained similar results, suggesting that CC combination with Gn regimen was less effective than GnRH agonist in producing more oocytes, but the rates of transplantation and pregnancy between the two regimens were comparable (18). In this study, we found that the pregnancy rate of PPOS regimen was significantly higher than mild stimulation regimen. Additionally, compared with the standard regimen, the number of oocytes retrieved and E2 level on hCG trigger day in mild stimulation regimen were significantly decreased. Serum E_2_ level reflects the quality of oocytes (19), and it has been shown to promote follicle production, upregulate gonadotropin receptors expression, repress the apoptosis of granulosa cell, and the number of dominant follicles is positively correlated with E_2_ levels (20). A previous study also showed that the mild stimulation regimen (CC/LE in combination with Gn) reduced E_2_ level on hCG trigger day and decreased the number of mature oocytes when compared with the standard regimen, thereby decreasing the chance of receiving live frozen embryos (21). Although these results suggested that ovulation induction efficiency and pregnancy outcomes of mild stimulation regimen were inferior to the other two regimens, the relatively low AMH of the CC/LE+Gn regimen group in our study might be the main reason for these biased outcomes.

Our findings also suggested that PPOS protocol effectively improved the IVF outcome of patients with endometrial disease without increasing the recurrence rate. On the premise that there was no significant difference in baseline characteristics, the PPOS regimen led to better-quality embryos compared with the standard regimen. It has been reported that the PPOS regimen, due to the advantage of the oral route, can prevent premature luteinizing hormone (LH) surges and lead to comparable oocyte retrieval and pregnancy outcomes compared to the GnRH-agonist short regimen (22). Dual triggering (low dose of hCG and GnRH agonist) for oocyte maturation, and the use of a freeze-all strategy to treat viable embryos almost completely avoids the occurrence of ovarian hyperstimulation syndrome (23). Furthermore, previous reviews showed that the recurrence rate of EEC/AEH after conservative treatment was reported to be in the range of 33.8–47% (24–26), and the mean duration of recurrence is 20–47.9 months (24, 25). In this study, 97 patients underwent diverse ovulation stimulation regimens including PPOS, CC/LE+Gn and standard regimens, and the recurrence rates were 16.22, 21.88, and 17.86%, respectively. Among them, 32.35% of patients canceled the FET cycle due to disease recurrence. Multivariate logistic regression analysis showed that the age, treatment time of endometrial lesions, E_2_ level on hCG trigger day and duration of Gn treatment were significantly associated with the recurrence of endometrial diseases, and the first two were found as the most reliable risk factors for the endometrial diseases recurrence after conservative therapy. Previous studies found that age is one of the main factors affecting the success of ovulation induction. Beginning after the age of 30, aging may have an effect on pregnancy outcomes, which may be due to a decrease in oocyte quality and endometrial receptivity (27, 28). In this study, we found that age at ovulation induction was closely related to the recurrence of endometrial diseases through univariate and multivariate logistic regression analysis. The risk of recurrence of endometrial diseases was increased with the age at ovulation induction. Thus, ovulation induction should likely be prescribed to younger women in order to avoid the risk of recurrence of endometrial diseases. Besides, the multivariate analysis showed that treatment time of endometrial lesions was a risk factor of endometrial disease recurrence, indicating that the duration of endometrial lesions treatment must be controlled to avoid disease recurrence, which could be detrimental for pregnancy outcomes.

This study is a retrospective study, so the limited number of patients and the inevitable heterogeneity of patients in different regimens restrict our conclusions. In order to verify the long-term beneficial effects of infertility treatment on EEC/AEH recovery, more large-scale studies involving a large number of pregnant women will be needed. In the meantime, we also need further prospective studies to confirm which ovulation induction protocol is effective and safe.

In summary, the age at ovulation induction and treatment time of endometrial lesions are two stable prognostic factors of the recurrence of endometrial diseases during IVF following fertility-sparing treatment. Moreover, the PPOS protocol is a feasible ovulation stimulation strategy without increasing the recurrence of endometrial diseases. Therefore, improvement of clinical pregnancy outcomes with the aid of PPOS regimen may improve long-term prognosis of patients undergoing IVF after conservative treatment of EEC or AEH.

## Data Availability Statement

The raw data supporting the conclusions of this article will be made available by the authors, without undue reservation.

## Ethics Statement

The studies involving human participants were reviewed and approved by Ethics Committees of Obstetrics and Gynecology Hospital of Fudan University (number JIAI E 2012-02). The patients/participants provided their written informed consent to participate in this study. Written informed consent was obtained from the individual(s) for the publication of any potentially identifiable images or data included in this article.

## Author Contributions

MY and XC contributed to the conception or design of the work and in revising the manuscript critically for important intellectual content. JC, YC, WF, XP, XS, and HC contributed the acquisition, analysis, or interpretation of data for the work. JC contributed in drafting the manuscript. All authors approved the final version of the manuscript prior to be published.

## Conflict of Interest

The authors declare that the research was conducted in the absence of any commercial or financial relationships that could be construed as a potential conflict of interest.

## Publisher's Note

All claims expressed in this article are solely those of the authors and do not necessarily represent those of their affiliated organizations, or those of the publisher, the editors and the reviewers. Any product that may be evaluated in this article, or claim that may be made by its manufacturer, is not guaranteed or endorsed by the publisher.
